# A combined genome-wide linkage and association approach to find susceptibility loci for platelet function phenotypes in European American and African American families with coronary artery disease

**DOI:** 10.1186/1755-8794-3-22

**Published:** 2010-06-07

**Authors:** Rasika A Mathias, Yoonhee Kim, Heejong Sung, Lisa R Yanek, VJ Mantese, J Enrique Hererra-Galeano, Ingo Ruczinski, Alexander F Wilson, Nauder Faraday, Lewis C Becker, Diane M Becker

**Affiliations:** 1 Genometrics Section, Inherited Disease Research Branch, National Human Genome Research Institute, National Institutes of Health, Baltimore, MD, USA; 2 School of Medicine, Johns Hopkins Medical Institutions, Baltimore, MD, USA; 3 Bloomberg School of Hygiene and Public Health, Johns Hopkins University, Baltimore, MD, USA

## Abstract

**Background:**

The inability of aspirin (ASA) to adequately suppress platelet aggregation is associated with future risk of coronary artery disease (CAD). Heritability studies of agonist-induced platelet function phenotypes suggest that genetic variation may be responsible for ASA responsiveness. In this study, we leverage independent information from genome-wide linkage and association data to determine loci controlling platelet phenotypes before and after treatment with ASA.

**Methods:**

Clinical data on 37 agonist-induced platelet function phenotypes were evaluated before and after a 2-week trial of ASA (81 mg/day) in 1231 European American and 846 African American healthy subjects with a family history of premature CAD. Principal component analysis was performed to minimize the number of independent factors underlying the covariance of these various phenotypes. Multi-point sib-pair based linkage analysis was performed using a microsatellite marker set, and single-SNP association tests were performed using markers from the Illumina 1 M genotyping chip from deCODE Genetics, Inc. All analyses were performed separately within each ethnic group.

**Results:**

Several genomic regions appear to be linked to ASA response factors: a 10 cM region in African Americans on chromosome 5q11.2 had several STRs with suggestive (p-value < 7 × 10^-4^) and significant (p-value < 2 × 10^-5^) linkage to post aspirin platelet response to ADP, and ten additional factors had suggestive evidence for linkage (p-value < 7 × 10^-4^) to thirteen genomic regions. All but one of these factors were aspirin *response *variables. While the strength of genome-wide SNP association signals for factors showing evidence for linkage is limited, especially at the strict thresholds of genome-wide criteria (N = 9 SNPs for 11 factors), more signals were considered significant when the association signal was weighted by evidence for linkage (N = 30 SNPs).

**Conclusions:**

Our study supports the hypothesis that platelet phenotypes in response to ASA likely have genetic control and the combined approach of linkage and association offers an alternative approach to prioritizing regions of interest for subsequent follow-up.

## Background

Platelet activation plays a critical role in atherothrombotic diseases such as acute myocardial infarction (MI) and stroke. Aspirin (acetylsalicylic acid [ASA]) is a mainstay of both primary and secondary prevention of MI and stroke [1]. ASA inhibits cyclooxygenase-1 (COX-1) and thromboxane-dependent platelet activation, which decreases the probability of acute thrombosis related proximally to cardiac and stroke events [2-4]. Large aspirin chemoprophylaxis trials, however, demonstrate that many individuals fail to achieve the expected protective benefits of therapy, presumably related to failure of ASA to adequately suppress platelet activation [5-7]. Residual platelet activation, from pathways directly and indirectly related to COX-1, is reported to be related to greater risk of MI and stroke in persons on aspirin therapy [8,9].

Previously, we have found evidence of moderate to high heritability of platelet function phenotypes, in both the presence [10] and absence of aspirin [11], suggesting that genetic variants contribute to differences among individuals in platelet activation and response to aspirin therapy. In this study, we take advantage of two alternative platforms of genotype data available, a linkage panel of microsatellite markers (STRs) and a genome-wide association study (GWAS) panel of single nucleotide polymorphisms (SNPs), in a combined linkage and association approach to search for loci controlling phenotypes in known platelet activation pathways.

## Methods

### Subjects

The study protocol was approved by the Johns Hopkins Institutional Review Board, and all subjects gave written informed consent. Subjects were identified from European American and African American families with premature coronary artery disease (CAD) through a proband with documented CAD prior to 60 years of age. Apparently healthy siblings of the probands, the adult offspring of both the probands and their siblings, and the coparents of the offspring were recruited for the **Gene**tic **St**udy of **A**spirin **R**esponsiveness (GeneSTAR). GeneSTAR was designed to examine genetic and environmental determinants of platelet function in response to low-dose aspirin therapy. Eligible subjects were ≥ 21 years of age and had no history of any coronary heart disease, thrombotic event, peripheral vascular disease, stroke, transient ischemic attacks, known derangement in hematologic profiles (aplastic anemia, Sickle Cell Disease, von Willebrand's Disease, Factor V Leiden), renal or hepatic failure, autoimmune diseases, glucocorticosteroid use, hemorrhagic event, measured blood pressure ≥ 180/105 mmHg or current pregnancy. Subjects were also excluded if they had an allergy or intolerance to ASA, baseline platelet count ≤ 100,000 or ≥ 500,000 cells/μL, hematocrit ≤ 30%, or white blood cell count ≥ 20,000 cells/μL. Anticoagulants, ASA, nonsteroidal anti-inflammatory agents, illicit drugs, were proscribed for 14 days before the baseline visit and during the study interval. Consumption of tea and coffee and flavenol-rich foods (egg, wine, grape juice, chocolate) and other dietary items known to affect platelet function (fish rich in omega-3 fatty acids and garlic) were prohibited for 24 hours and smoking for 12 hours prior to assessments. Subjects with zero aggregation of platelets to arachidonic acid in both whole blood and platelet-rich plasma (PRP) at the baseline visit and questionable adherence to the list of proscribed substances, were omitted from the study. At the end of the first screening visit, eligible subjects were given a supply of 81-mg ASA tablets and instructed to take 1 pill each day for 14 days. Adherence to ASA use during the study was assessed at the post-ASA visit using pill counts and an adapted standardized medication adherence questionnaire [12].

### Laboratory Measurements

Blood for the measurement of platelet function in whole blood (WB) and platelet rich plasma (PRP) was obtained by venipuncture at the same time of day at baseline and following 14 days of ASA therapy. Baseline and post-ASA measures included aggregation, slope, and lag time in response to selected doses of 4 known platelet agonists, and urinary excretion of the thromboxane B2 metabolite 11- dehydrothromboxane B2 (Tx-M). Optical aggregation was measured in PRP in a PAP-4 aggregometer (Horsham, PA) after samples were stimulated with arachidonic acid (1.6 mmol/L, collagen (1, 2, 5 μg/mL), ADP (2, 10 mmol/L), and epinephrine (2, 10 μmol/L). Whole blood impedance aggregometry was measured in a Chrono-Log dual-channel lumiaggregometer (Havertown, PA) after samples were stimulated with arachidonic acid (0.5 mmol/L), collagen (1, 5 μg/mL), and ADP (10 μmol/L). Platelet function under shear stress was determined by the platelet function analyzer (PFA) test (PFA-100, Dade- Behring, Newark, DE). Whole blood was loaded into standard proprietary cartridges (Dade-Behring) containing collagen and epinephrine, and aperture closure time was recorded in seconds (maximum of 300 seconds).

Plasma fibrinogen was assayed in the Johns Hopkins Clinical Coagulation Laboratory and von Willebrand factor (vWF) was measured using enzyme-linked immunosorbent assays (DiaPharma, West Chester, OH).

Urine was collected at the same time of day for pre- and post-ASA measurements of Tx-M using an enzyme-linked immunosorbent assay (Cayman Chemical Co., Ann Arbor, MI) and values were normalized to urinary creatinine.

### Cardiac Risk Factor Evaluation

Blood pressure was measured according to methods of the American Heart Association and hypertension was defined as the average of 4 resting blood pressures ≥ 140/90 mm Hg and/or the taking of antihypertensive medications. Current smoking was defined as any reported cigarette smoking within the past 30 days and nonsmoking status was validated by exhaled CO levels < 8 ppm on the average of 2 measurements. Height and weight were measured and body mass index was calculated as the weight (kg)/height squared (m^2^). Plasma glucose, total cholesterol, triglyceride, and HDL-cholesterol levels were measured after patients had fasted for 12 hours overnight. LDL cholesterol was estimated using the Friedewald equation. Diabetes was defined as self-reported diabetes with the use of diabetes medications, or measured glucose levels ≥ 126 mg/dl.

### Genotyping

Microsatellite genotyping was performed at deCODE Genetics, Inc. with the standard deCODE 550 STR marker set (average spacing = 8 cM). There were 574 successfully released STR markers with an overall duplicate error rate (3 CEPH sample duplicates on each of 18 plates) of 0.1% and an overall Mendelian error rate of 2% on 99.7% of samples. The STR genotyping data were checked for Mendelian inconsistencies using PEDCHECK [13], and all persons with inconsistencies were removed prior to analysis. The most likely relationship between pairs of relatives was inferred using RELCHECK [14], and these were used to verify self-reported relationships. SIBPAIR (v 0.99.9) was used to calculate allele frequencies where the contribution of each pedigree is weighted by the number of founders it contains.

SNP genotyping was performed at deCODE Genetics, Inc. using the Human 1Mv1_C array from Illumina, Inc. and 1,044,977 markers were released with an average call rate per sample of 99.65% and an overall missing data rate of 0.35%. Using 25 duplicate pairs of CEPH samples, the reproducibility rate was >99.95% for all duplicate pairs. Analyses at deCODE Genetics revealed Mendelian errors (> 5%) in 14 samples, which were eliminated from further analysis. Finally, 9 samples with gender discrepancies and an additional 3,427,500 inconsistency calls over all SNPs were eliminated from the final data prior to analyses. While all markers were analyzed, SNPs were flagged for closer examination where minor allele frequency was low (2%) and/or deviation from Hardy Weinberg Equilibrium was severe (p-value < 10^-6^).

### Statistical Methods

Given the large number of potentially different biologically-related platelet function cascades examined [10], principal components analysis (PCA) was used to define a set of independent factors that explained a large proportion of the phenotypic co-variance. PCA was run separately for European Americans and African Americans within each of three major groups of outcome phenotypes: (1) baseline, representing native platelet function, (2) post-aspirin, representing platelet function measures after 2 weeks of daily aspirin, and (3) post-aspirin platelet function adjusted for pre-aspirin platelet function, representing the change attributable to aspirin, or aspirin responsiveness. Prior to PCA, all platelet variables were first adjusted for age and sex, levels of LDL cholesterol, fibrinogen, and body mass index, and for the presence of diabetes, hypertension, and current smoking using linear regression models. For PFA test only, von Willebrand's factor was included in the adjustment. Following adjustment for covariates, there were 37 pre-ASA platelet function variables, 32 post-ASA variables, and 27 post-adjusted for pre-ASA variables within each race with distributions that were adequate to enable calculation of accurate Z-scores. All Z-scored variables were then used for PCA in PROC Factor in SAS version 9.1 implementing orthogonal varimax rotations. Eight components with Eigenvalues > 1 were identified for the pre-ASA variables, post ASA and post-adjusted for pre-ASA variables within each race. The components were labeled by their primary platelet phenotypic variables, defined as loadings > 0.4. These final PCA components were used in the tests for linkage and additional association analyses described below.

Linkage analysis was performed with the Haseman-Elston regression approach [15] in SAGE (v 5.1.0) for each principal component and each STR for each ethnic group separately. In these analyses the traditional Haseman-Elston analysis for a quantitative trait (i.e. the regression of the squared traits difference on the IBD estimate) was performed on full sib relationships under a multipoint approach. This approach has been shown to be adequately robust and powered even for sample sizes with less than 300 sib-pairs [16]. We used p-values defined by Lander and Kruglyak [17] to represent specific LOD score thresholds: (1) suggestive linkage with p value = 0.00074 and corresponding LOD = 2.2; (2) significant linkage with p-value = 0.00002 and corresponding LOD = 3.6; and (3) highly significant linkage with p- value = 0.0000003 and corresponding LOD = 5.4.

Associations between SNPs on the GWAS panel and factors that had evidence for linkage based on the analyses described above were tested using linear mixed effects models (LME). In the formulation of the LME model, SNP genotypes were included as fixed effects setting the genotypes to be additive in effect, and family identification number was included in the random effects (essentially treating the correlations between all pairs of individuals in the family as equal). We tested whether the additive effects of each SNP was different from zero. The LME in SAS (v. 9.1.3 for Linux OS) was applied with PROC MIXED using the option for EMPIRICAL variance.

In an attempt to offset the diminished power to detect association that arises as a result of multiple testing issues inherent in genomic searches and to determine the best set of loci to pursue in further follow-up studies (i.e. to control false positive association signals) in the absence of external replication data, we used the False Discovery Rate (FDR) approach proposed by Roeder et al [18]. This new weighted FDR methodology involves weighting the association test p-values on the basis of prior data derived from linkage. A combined map was obtained; interpolating the STRs with the SNP map by assigning a physical location (in Mb) to the mid-point of the STR. Using p-values from the linkage scan at these assigned physical locations, continuous linkage traces were derived from the standard normal cumulative distribution and used to weight the association p-values prior to the calculation of the FDR threshold. In this analysis we used Storey's FDR approach [19] which can be more powerful than that proposed by Benjamini and Hochberg [20] under the same error rate (here, alpha = 0.05).

## Results

Clinical data on agonist-induced platelet function, PFA, and Tx-M phenotypes were evaluated in healthy subjects with a family history of premature CAD before and after a 2-week trial of ASA (81 mg/day). There were 1231 European American subjects (45% male) from 398 families, and 846 African Americans (38% male) from 243 families. Linkage analysis was performed using 257 sib-pairs in European Americans and 158 in African Americans. The clinical characteristics of the population are presented on Table 1.

**Table 1:** Clinical Characteristics of subjects.

Characteristic	European Americans (n = 1231)	African Americans (n = 846)
Female sex (%)	55.24	61.58
Diabetic (%)	5.71	12.22
Hypertensive (%)	26.28	40.62
Current smoking (%)	23.09	30.26
Age (years, mean ± SD)	44.52 ± 13.2	43.29 ± 12.4
Total cholesterol (mg/dl, mean ± SD)	203.94 ± 41.0	196.68 ± 43.4
HDL cholesterol (mg/dl, mean ± SD)	50.91 ± 14.6	54.93 ± 16.1
Triglycerides (mg/dl, mean ± SD)	141.28 ± 83.5	107.36 ± 71.6
LDL cholesterol (mg/dl, mean ± SD)	125.15 ± 37.1	120.49 ± 38.5
Glucose (mg/dl, mean ± SD)	94.08 ± 20.7	98.29 ± 36.1
Fibrinogen (mg/dl, mean ± SD)	374.41 ± 111.4	416.67 ± 127.9
vWF (%normal, mean ± SD)	87.60 ± 58.6	87.24 ± 53.8
Blood pressure (mmHg systolic/diastolic, mean ± SD)	118.25 ± 14.9/75.69 ± 9.6	123.36 ± 18.4/79.32 ± 11.1

**Figure 1:**
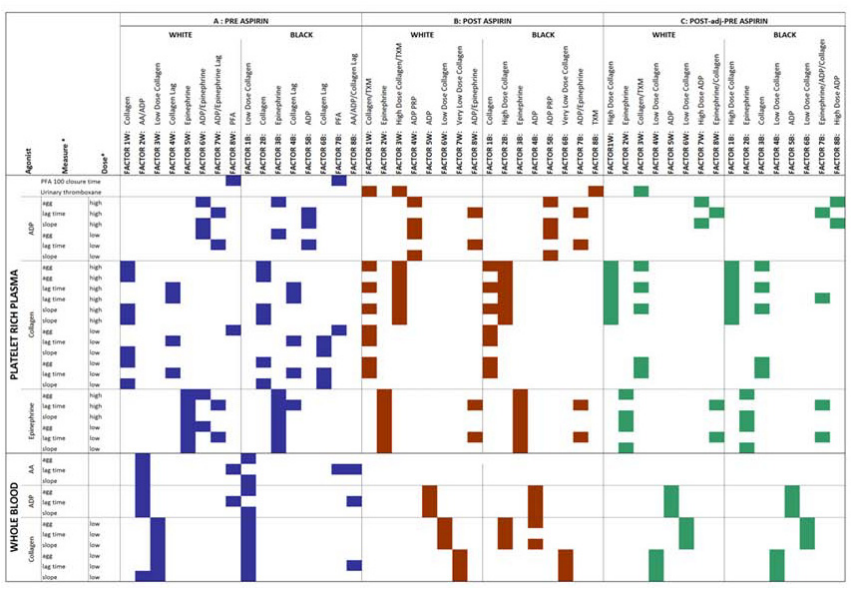
**Summary of the factors derived from the Principal Components Analysis (PCA) in European Americans and African Americans on 37 phenotypes measured pre-aspirin (A), post aspirin (B) and post-adjusted-for-pre aspirin (C)**. Each column represents a single factor and the color block indicates a phenotype with PCA loading > 0.4 for the specific factor. (* = where applicable).

Figure 1 illustrates the principal components derived from the individual platelet phenotypes for the three major phenotype groups (pre-ASA, post-ASA, post-adjusted for pre-ASA). For each principal component, the phenotypes that had factor loadings > 0.4 are labeled in color, and several key points emerge. While there are differences in the exact phenotypes and their precise loadings, the identity related to an underlying biological pathway and the order of the principal components was similar for European Americans and African Americans for each of the three major phenotype groups. The phenotypes appear to cluster largely by agonist, followed by dose and/or nature of the measure (i.e. slope vs. lag vs. aggregation) in loading onto factors. In fact, all of the major phenotype groups in both ethnicities contained principal components for collagen aggregation in PRP, epinephrine induced aggregation (EPI), ADP induced aggregation in PRP (PRPADP), and lag time to aggregation in PRP.

Most of the principal components were heritable, as shown in Table 2. The great majority of heritability estimates ranged from 0.26 - 0.70 in European Americans and 0.29 - 0.84 in African Americans. A few, however, appeared to have either low estimates of heritability (e.g., < 0.10) or estimates with large standard errors (Table 2).

**Table 2:** Heritability Estimates of factors in African American and European American pedigrees.

	White			African American		
	**Factor**	**Estimate**	**S.E.**	**Factor**	**Estimate**	**S.E**.

**Post Aspirin Factors**	1W: Collagen/TXM	0.50	0.09	1B: Collagen	0.36	0.14
	2W: Epinephrine	0.61	0.09	2B: High Dose Collagen	0.84	0.13
	3W: High Dose Collagen/TXM	0.47	0.10	3B: Epinephrine	0.46	0.15
	4W: ADP PRP	0.34	0.09	4B: ADP	0.29	0.14
	5W: ADP	0.54	0.09	5B: ADP PRP	0	
	6W: Low Dose Collagen	0.40	0.09	6B: Very Low Dose Collagen	0.32	0.15
	7W: Very Low Dose Collagen	0.31	0.09	7B: ADP/Epinephrine	0.33	0.17
	8W: ADP/Epinephrine	0.05	0.09*	8B: TXM	0.51	0.15

**Post-adjusted-for-pre Aspirin Factors**	1W: High Dose Collagen	0.67	0.11	1B: High Dose Collagen	0.51	0.17
	2W: Epinephrine	0.41	0.10	2B: Epinephrine	0.31	0.13
	3W: Collagen/TXM	0.40	0.10	3B: Collagen	0.43	0.20
	4W: Low Dose Collagen	0.18	0.11*	4B: Low Dose Collagen	0.08	0.12*
	5W: ADP	0.27	0.10	5B: ADP	0.15	0.13*
	6W: Low Dose Collagen	0.18	0.10*	6B: Low Dose Collagen	0.34	0.17
	7W: High Dose ADP	0.21	0.11*	7B: Epinephrine/ADP/Collagen	0.31	0.15
	8W: Epinephrine/Collagen	0.26	0.10	8B: High Dose ADP	0	

**Pre Aspirin Factors**	1W: Collagen	0.19	0.12*	1B: Low Dose Collagen	0.58	0.17
	2W: AA/ADP	0.61	0.10	2B: Collagen	0.19	0.22*
	3W: Low Dose Collagen	0.70	0.10	3B: Epinephrine	0.55	0.19
	4W: Collagen Lag	0.37	0.11	4B: Collagen Lag	0.1	0.13*
	5W: Epinephrine	0.51	0.10	5B: ADP	0.03	0.23*
	6W: ADP/Epinephrine	0.30	0.10	6B: Collagen Lag	0.22	0.15*
	7W: ADP/Epinephrine Lag	0.07	0.10*	7B: PFA	0.12	0.18*
	8W: PFA	0.20	0.11*	8B: AA/ADP/Collagen Lag	0.58	0.17

* = not statistically significant at α = 0.05

Tables 3 and 4 summarize the linkage results in European American and African American pedigrees, respectively, presenting all signals with at least suggestive evidence for linkage (p-value < 7 × 10^-4^). At thresholds of significant linkage (p-value < 2 × 10^-5^) a ~10 cM region on chromosome 5q11.2 appears to be linked to a post-ASA principal component representing aggregation to ADP in whole blood (Factor 4B (POST): ADP) in African Americans; no significant evidence was noted in European Americans. In addition, a total of eleven factors, five in European Americans and six in African Americans, appear to have suggestive evidence for linkage across fourteen regions in the genome. The single most striking feature of these results in Tables 3 and 4 is that the evidence for linkage is observed largely for the post- and post-adjusted-for-pre-ASA factors, i.e. platelet *response *to aspirin intervention therapy.

**Table 3:** Summary of multipoint linkage signals in European American pedigrees.

Significance Criteria	Chr	Map Position	Marker	Factor
P < 0.00002	-	-	-	

P < 0.00074	3	89.04039	D3S1600	2W(POST):Epinephrine
	4	62.104	D4S405	3W(POST):High Dose Collagen/TXM
	4	67.63	D4S396	3W(POST):High Dose Collagen/TXM
	4	71.03	D4S428	3W(POST):High Dose Collagen/TXM
	5	203.456	D5S469	5W(PP):ADP
	6	174.4557	D6S1581	3W(PP):Collagen/TXM
	11	126.83	D11S4089	2W(POST):Epinephrine
	15	43.142	D15S146	7W(PP):High Dose ADP
	15	52.712	D15S1016	7W(PP):High Dose ADP

POST = Post-ASA Factor, PRE = Pre-ASA Factor, PP = Post-adjusted-for-pre-ASA Factor, Chr = chromosome

Significance criteria as defined by Lander and Kruglyak (1995): suggestive linkage (p-value < 7 × 10^-4^) and significant linkage (p-value < 2 × 10^-5^).

**Table 4:** Summary of multipoint linkage signals in African American pedigrees.

Significance Criteria	Chr	Map position	Marker	Factor
P < 0.00002	5	71.143	D5S628	4B(POST):ADP
	5	80.82	D5S2072	4B(POST):ADP

P < 0.00074	1	160.45	D1S484	2B(PRE):Collagen
	1	216.774	D1S245	4B(POST):ADP
	1	217.215	D1S205	4B(POST):ADP
	2	78.417	D2S2156	5B(POST):ADP PRP
	5	83.18814	D5S1967	4B(POST):ADP
	8	69.341	D8S1737	4B(POST):ADP
	11	154.04129	D11S969	4B(POST):ADP
	17	102.596	D17S940	5B(PP): ADP
	18	21.744	D18S967	7B(POST): ADP/Epinephrine
	19	99.84163	D19S572	4B(PRE):Collagen Lag

POST = Post-ASA Factor, PRE = Pre-ASA Factor, PP = Post-adjusted-for-pre-ASA Factor, Chr = chromosome

Significance criteria as defined by Lander and Kruglyak (1995): suggestive linkage (p-value < 7 × 10^-4^) and significant linkage (p-value < 2 × 10^-5^).

**Figure 2:**
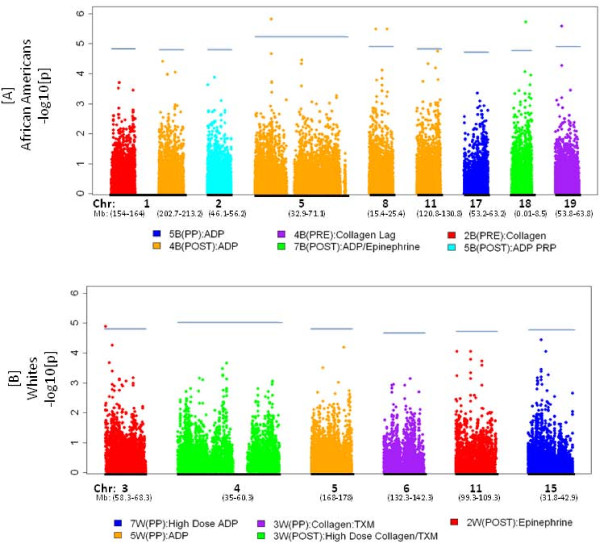
**Tests for association under peak areas of linkage in African American [A] and European American [B] pedigrees selected for increased CAD risk**. Significance plotted as -log10(p-value) against chromosomal distance for each factor with suggestive or significant linkage to the specific chromosome region from Tables 3 and 4. Region-specific Bonferroni thresholds are indicated by horizontal blue lines specific to each region (defined as 0.05/n; where n = number of SNPs tested in a region of +/- 5 Mb on either side of the linked STR).

Figure 2 illustrates the evidence for association in these regions of suggestive and significant linkage, specifically focusing on a 10 cM (i.e. ~10 Mb) region centered on each STR marker that had a linkage p-value < 7 × 10^-4 ^(see Tables 3 and 4), and restricted to those specific eleven factors linked to these regions. While there does not appear to be significant association signal in these regions at full genome-wide association criteria (p-value < 10^-8^), seven SNPs reach a *region-specific *Bonferroni threshold (p < 0.05/n, where n = number of SNPs in each specific region of linkage) as presented in Additional file 1.

**Figure 3:**
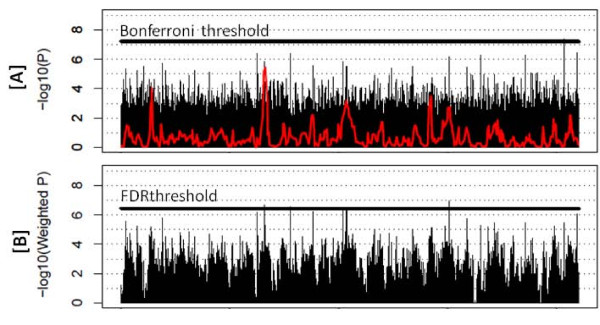
**Genome wide tests for association for Factor 4B(POST):ADP in African American pedigrees illustrating [A] P-values from linear mixed models along with the linkage traces in red, and [B] linkage weighted P-values**.

Full genome-wide tests for association in these six factors in African Americans and five in European Americans with suggestive evidence for linkage (p-value < 7 × 10^-4 ^in Tables 3 and 4) reveal little signal that meets the strict Bonferroni GWAS criteria (p-value < 10^-8^). Specifically, 9 SNPs met these criteria as indicated in Table 5. In the absence of external validation of our association results from replication studies, we maximize information available in our data to determine the best set of signals to take forward to further follow-up studies. We used weighted FDR methods for the association tests that take into account linkage information and we observed an increase in signals now considered to be significant at GWAS criteria relying on FDR methodology. With this approach, 30 signals now meet these new FDR-defined criteria as indicated in Table 5. To illustrate the added benefit of this combined approach Figure 3 depicts the un-weighted GWAS p-values along with the multi-point linkage signal (in red) in Panel A alongside the newly linkage-weighted p-values and new significance threshold in Panel B for factor 4B(POST):ADP. It is clear that the signals of association are not restricted to regions of peak linkage, but are in fact in regions with modest linkage. Of the nine signals detected by the stringent Bonferroni un-weighted approach, eight are significant with the linkage weights applied and one signal appeared to be no longer of significance when the linkage evidence was considered.

**Table 5:** Significant genome wide association signals defined at Bonferroni thresholds for association tests (P) and false discovery rate controlled linkage-weighted P values (WP) for the eleven factors with linkage evidence.

SNP	Closest Gene	4B(PRE): CollagenLag		4B(POST): ADP		5B(POST): ADPPRP		5B(PP): ADP		7B(POST): ADP/Epinephrine		2W(POST): Epinephrine		3W(POST): HighDoseCollagen/TXM		3W(PP): Collagen/TXM		7W(PP): HighDoseADP	
		P	WP	P	WP	P	WP	P	WP	P	WP	P	WP	P	WP	P	WP	P	WP
rs6796806														9.3E-09	3.1E-09				
rs6764798														4.1E-08	1.3E-08				
rs1436634	MME															2.3E-08	8.9E-09		
rs1994882					2.14E-07														
rs7712189					2.14E-07														
rs2112172									1E-07										
rs6917811					2.55E-07														
rs1534446	PIP3-E														5.9E-08				
rs13272289					2.96E-07														
rs16925150									1.1E-07										
rs10116901	GLIS3															4.7E-10	6.6E-08		
rs1485187																1.0E-08	4.9E-09		
rs7045878											3.1E-08								
rs1676780															2.7E-07				
rs1776122															3.1E-07				
rs6583759													9.1E-08						
rs7079291												5.5E-09	2.5E-08						
rs11032080																			2.1E-07
rs12365876																			2.5E-07
rs11024665	LDHAL6A								7.7E-08										
rs936368													1.1E-07						
rs1458072													1.8E-07						
rs2971589		1.5E-08	5.2E-08																
rs17029861	ANKS1B				1.1E-07														
rs2373201	ANKS1B				2.1E-07														
rs16940502						5.5E-09	3.8E-07												
rs8104319																			1.3E-07
rs3810340																			1.3E-07
rs4281830																			1.3E-07
rs917652																			1.6E-07
rs6025934				4.3E-08															

Factors 5W(PP):ADP and 2B(PRE):Collagen do not have any SNPs that reach either significance threshold.

## Discussion

To date only specific agonists or single pathway platelet function phenotypes have been examined offering little insight into the potentially complex interplay among platelet function cascades. The GeneSTAR study is the most comprehensive assessment of platelet response to aspirin done to date and given the cost, labor intensiveness, and methodologic difficulty in measuring platelet function across multiple agonists and doses in a large sample size, it is unlikely that another study of this magnitude will exist to support external validation of association signals. The PCA-derived traits represent novel variables that take into account the naturally occurring correlations among important platelet variables. While the PCA-derived phenotypic values are not in and of themselves intuitively informative, they enable the identification of possibly important loci for more integrated platelet phenotypes that represent baseline platelet function or true global responses to aspirin.

We noted linkage in fourteen regions of the genome for eleven factors; interestingly, only one single linkage signal was found for factors representing baseline platelet function. The reasons for this are uncertain, but there is likely to be a greater variability in phenotype in native platelets as compared with measurements following ASA administration. ASA inhibits thromboxane-related aggregation, a pathway which augments aggregation to a variable extent, in a positive feedback loop initiated by other agonists, such as collagen. By removing variability in platelet function associated with the thromboxane pathway, ASA treatment may increase the ability to detect genotype-phenotype association signals related to the remaining platelet activation pathways.

Genome-wide association studies rapidly emerged as the leading tool in the identification of disease susceptibility loci in the recent past [21] and have proved successful in mapping novel and previously not implicated loci for a multitude of diseases [22-24]. However, these studies have largely been successful in mapping trait/disease-associated SNPs that are common (with median risk allele frequency shown to be 36%) and having only modest effect sizes (median odds ratio OR 1.33) [25] that together only account for a small fraction of the total risk/variance of the diseases/traits [26]. With much of the genetic variation in these traits as yet left to discover, attention has been focused on issues of increased sample size, incorporating more than main effects of SNPs (i.e. accounting for gene*environment and gene*gene interactions), capturing variation not assessed in commercial GWAS arrays (i.e. low frequency variants and copy number variants) and region/gene-based signals rather than pure SNP-SNP replication [27]. Another approach would be to leverage prior information in the evaluation and prioritization of genome wide association signal [18]. Here, the availability of linkage and association information from two independent methods of analysis provides this unique opportunity, allowing us to prioritize signals for future follow-up based on the combined signal from both approaches.

While many significant association signals are intergenic, several genes are identified in Table 5, some of which are related to pathways known to be involved with platelet function. One such example in European Americans is *MME *(a membrane metalloendopeptidase) on chromosome 3q21-27, associated with ASA response to collagen induced aggregation in PRP).

## Conclusions

Given that GeneSTAR is the most comprehensive assessment of platelet response to aspirin done to date, this also leads to two general weaknesses: our limited sample size for linkage and the lack of external replication. In light of these two limitations, we have implemented several novel approaches to best search for susceptibility loci, implementing the principal component approach to first find factors that represent the underlying biological correlation between the multitude of measured phenotypes and the combined approaches of linkage and association with two sets of marker data. In conclusion, this study is unique in its ability to identify loci controlling platelet response to aspirin intervention in both European American and African American families identified to be at risk for CAD, and our combined novel approaches have yielded several loci that we believe worthy of further follow up.

## Abbreviations

ASA: Aspirin; CAD: coronary artery disease; STRs: microsatellite markers; GWAS: genome-wide association study; SNP: single nucleotide polymorphism; PRP: platelet-rich plasma; WB: whole blood; vWF: von Willebrand factor; PCA: principal components analysis; LME: linear mixed effects models; FDR: False Discovery Rate.

## Competing interests

The authors declare that they have no competing interests.

## Authors' contributions

Conceived and designed the linkage and genome-wide association study: NF, LCB, DMB. Conceived and designed the research questions proposed within this manuscript: RAM, YK, IR, AFW, NF, LCB, DMB. Conceived and designed the phenotyping and acquired the phenotype data: LRY, EHG, IR, NF, LCB, DMB. Analyzed the data: RAM, YK, HS, VM. Wrote the first draft of the paper: RAM, YK. Contributed to the manuscript editing, revising, and final approval of the version to be published: RAM, YK, HS, LRY, VM, EHG, IR, AFW, NF, LCB, DMB.

## Pre-publication history

The pre-publication history for this paper can be accessed here:

http://www.biomedcentral.com/1755-8794/3/22/prepub

## Supplementary Material

Additional file 1 **Table S1**. Association signals under linkage peaks in Figure 2 that meet region-specific Bonferroni threshold criteria. Click here for file
