# Supplementary material for: A combined genome-wide linkage and association approach to find susceptibility loci for platelet function phenotypes in European American and African American families with coronary artery disease
Source: BMC Med Genomics. 2010 Jun 7;3:22. doi: 10.1186/1755-8794-3-22 (PMC2890666; doi:10.1186/1755-8794-3-22)
Supplement: Additional file 1 — Table S1. Association signals under linkage peaks in Figure 2 that meet region-specific Bonferroni threshold criteria. [file 1755-8794-3-22-S1.DOCX]

**Table S1:** Association signals under linkage peaks in Figure 2 that meet region-specific Bonferroni threshold criteria.

| **SNP** | **Chromosome** | **Position** | **Function** | **2W(POST):Epinephrine** | **4B(POST):ADP** | | **4B(PRE):Collagen Lag** | **7B(POST): ADP/Epinephrine** |
| --- | --- | --- | --- | --- | --- | --- | --- | --- |
| **Region-specific Bonferroni threshold** | | | | **1.43E-05** | **5.34E-06** | **1.13E-05** | **1.20E-05** | **1.56E-05** |
| rs9862378 | 3 | 58299434 | PXK (intron) | 1.26E-05 |  |  |  |  |
| rs1994882 | 5 | 39841921 |  |  | 1.48E-06 |  |  |  |
| rs7712189 | 5 | 39843346 |  |  | 1.48E-06 |  |  |  |
| rs10086768 | 8 | 18256634 |  |  |  | 3.21E-06 |  |  |
| rs1001792 | 8 | 22957147 | TNFRSF10B (intron) |  |  | 3.17E-06 |  |  |
| rs505145 | 18 | 5986517 | L3MBTL4 (intron) |  |  |  |  | 1.87E-06 |
| rs273628 | 19 | 56497130 |  |  |  |  | 2.58E-06 |  |
